# The effects of toe grip training on physical performance and cognitive function of nursing home residents

**DOI:** 10.1186/s40101-019-0202-5

**Published:** 2019-08-28

**Authors:** Ryota Tsuyuguchi, Satoshi Kurose, Takayuki Seto, Nana Takao, Aya Fujii, Hiromi Tsutsumi, Shingo Otsuki, Yutaka Kimura

**Affiliations:** 1 Department of Health Science, Graduate School of Medicine, Kansai Medical University, 2-5-1 Shinmachi, Hirakata, Osaka 573-1010 Japan; 2 Department of Health Science, Kansai Medical University, 2-5-1 Shinmachi, Hirakata, Osaka 573-1010 Japan; 3 Faculty of Sport and Health Sciences, Osaka Sangyo University, 3-1-1 Nakagaito, Daito, Osaka 574-8530 Japan

**Keywords:** Cognitive function, Nursing home residents, Toe grip strength, Toe grip training

## Abstract

**Background:**

Toe grip-related training requires individuals to actively exercise muscles that are not frequently used; therefore, it may improve not only toe grip strength but also cognitive function. The purpose of this study was to examine the effects of toe grip-related training on predictors of physical performance and cognitive function in nursing home residents.

**Methods:**

A total of 35 nursing home residents (35 left and 35 right feet; mean age, 82.1 ± 7.9 years) were included in this study. The participants were divided into two groups: a training group and a control group. The Mini-Mental State Examination (MMSE) was used to assess the cognitive function of the participants, and the Fall Risk Index (FRI) was used to evaluate the risk of falls. Toe grip-related physical function was also assessed. Baseline endpoints were evaluated and the effects of toe grip-related training were examined following a 12-week training intervention.

**Results:**

The training group showed significant improvements in MMSE score, FRI score, toe grip strength, and the toe skill (TS) test; however, the control group did not show these changes. The training group showed significant increases in Δ MMSE, Δ toe grip strength, and Δ TS (right foot) than the control group. Stepwise regression analysis revealed that Δ toe grip strength is an independent factor of Δ MMSE.

**Conclusions:**

Toe grip training improves not only toe grip strength itself, but also cognitive function. Furthermore, change in toe grip strength was an independent factor of change in MMSE in those populations.

**Trial registration:**

UMIN, UMIN000027437. Registered on 26 May 2017.

## Background

Toe grip strength has recently begun to gain attention. Toe gripping is performed using the flexor hallucis brevis, flexor hallucis longus, lumbricals, flexor digitorum brevis, and flexor digitorum longus muscles. Toe grip strength is related to both gait speed and the ability to balance on one leg and plays an important role in controlling fall-related lower limb movements [1–3]. In addition, muscle activity around the ankle joint is supported by toe grip strength, because the crural muscles achieved 30–50% integrated electromyography of the maximum voluntary contraction during exertion of maximal foot-ripping strength force in all positions [4]. The action of the toe is believed to be a strong predictor of falls because it plays an important role in balance control. In our previous studies, logistic regression analysis performed in community-dwelling elderly people, with presence or absence of falls as the dependent variables, identified toe grip strength as an independent risk factor for falls [5]. Recently, a device has been developed to measure toe grip strength. Toe grip strength could be a useful measure to predict the likelihood of falls.

As Japan is becoming a “super-aged” society, the number of nursing home residents has increased. The incidence of falls is significantly higher in nursing home residents than in community-dwelling elderly people. While the rate of community-dwelling elderly people who fall more than once per year is about 30% [6–8], that of nursing home residents is 30–56% [9–11]. The incidence of fall-related bone fractures is 0.5–8.4%, and 95% of fractures in nursing home residents are caused by a fall [12, 13]. Falls and fractures reduce the quality of life (QoL) of individuals because of severe physical and psychological distress and functional impairment; therefore, prevention of falls is extremely important in those populations. Although residents in a nursing or a care home tend to have high levels of activities of daily living (ADL), it is important to prevent a decline in the QoL of these individuals, caused by low levels of physical activity. In addition, it has been reported that dementia is related to falls [14]. Thus, a comprehensive intervention, not only for toe grip strength, but also for declining cognitive function and other related factors is required. Toe grip-related training requires individuals to actively exercise muscles that are not frequently used; therefore, it may improve not only toe grip strength but also cognitive function [15]. Previous studies have found that there is a limitation to the level of improvement in cognitive function that can be achieved with simple exercise [16]. The simple exercise structured moderate-intensity physical activity program that included walking, resistance training, and flexibility exercises or a health education program of educational workshops and upper-extremity stretching. The dorsal and ventral premotor cortexes are activated by performing multiple tasks requiring movement of the fingers [17]. However, no studies have evaluated both toe grip strength and cognitive function in nursing home residents. Thus, a study that develops training to improve toe grip strength and demonstrates the importance of toe grip strength in the prevention of falls and dementia is clinically significant.

We hypothesized that toe grip-related training would improve toe grip strength and cognitive function in nursing home residents. The purpose of this study was to examine the effects of toe grip-related training on predictors of physical performance (e.g., toe grip strength) and cognitive function in nursing home residents. The results of this intervention study will contribute to the aging society for the improvement of the current situation in the elderly populations.

## Methods

### Participants

A total of 35 nursing home residents (35 left and 35 right feet; mean age, 82.1 ± 7.9 years) from Joyo City, Kyoto, Japan, were included in this study. This facility has a maximum capacity of 50 people. The rate of certification of long-term care insurance was 60.0% (from support level 1 to care level 2). In the long-term care insurance system of Japan, when persons require long-term care due to being bedridden or having dementia (a condition of need for long-term care) or support for daily life such as housework and dressing (a condition of need for support), they are eligible to receive nursing-care services. It is determined whether a person is in need for long-term care or in need for support, and if long-term care is needed, the level of nursing-care requirement is determined by the long-term care approval board located in municipalities and by insurers. The characteristics of nursing home residents are shown in Table 1.

**Table 1:** Characteristics of nursing home residents at baseline

	All	Training group	Control group	*p* value
Age (year)	82.1 ± 7.9	80.7 ± 7.6	85.2 ± 8.1	0.136
Gender (male/female)	12/23	10/14	2/9	0.174
Height (cm)	148.4 ± 9.5	149.0 ± 9.7	147.2 ± 9.4	0.609
Weight (kg)	48.0 ± 9.3	50.2 ± 8.4	43.3 ± 9.6	0.051
Body mass index (kg/m^2^)	21.7 ± 3.0	22.6 ± 2.8	19.8 ± 2.8	0.014
Category of conditions requiring long-term care				
None; *n*, (%)	14, (40.0%)	10, (41.7%)	4, (36.4%)	0.413
Support level 1; *n*, (%)	4, (11.4%)	3, (12.5%)	1, (9.1%)	
Support level 2; *n*, (%)	5, (14.3%)	5, (20.8%)	0, (0.0%)	
Care level 1; *n*, (%)	9, (25.7%)	4, (16.7%)	5, (45.5%)	
Care level 2; *n*, (%)	3, (8.6%)	2, (8.3%)	1, (9.1%)	
Medical history				
Obesity; *n*, (%)	4, (11.4%)	4, (16.7%)	0, (0.0%)	0.150
Hypertension; *n*, (%)	22, (62.9%)	17, (70.8%)	5, (45.5%)	0.149
Diabetes mellitus; *n*, (%)	6, (17.1%)	6, (25.0%)	0, (0.0%)	0.068
Heart disease; *n*, (%)	5, (14.3%)	3, (12.5%)	2, (18.2%)	0.656
Cerebrovascular disease; *n*, (%)	2, (5.7%)	1, (4.2%)	1, (9.1%)	0.560
Orthopedic disease; *n*, (%)	2, (5.7%)	1, (4.2%)	0, (0.0%)	0.492

Data are presented as mean ± SD

The statistical analysis used in the Mann-Whitney *U* test and the chi-square test

The condition of need for support, including levels 1 and 2, is a condition in which a person is able to live almost by oneself but requires certain support for maintaining/improving physical activity as prevention against developing to the condition of need for long-term care. The condition of need for long-term care, including levels 1–5, is a condition requiring long-term care in which a person is in poor physical condition and has difficulty living alone

The participants were divided into two groups, according to whether they wished to participate in the health exercise program or not: the training group (the group that received toe grip-related training, *n* = 24) and the control group (the group was instructed not to make any changes in their usual activities, *n* = 11). Nursing homes are facilities specified by the act on social welfare for the elderly aged ≥ 60 years with low ADL independence and without a family or family support. Individuals with a visible toe deformity and those with cerebral palsy or those who have difficulty living by themselves independently were excluded from this study. The inclusion criteria required participants to be able to undergo the measurements required by the study protocol. Toe grip-related training was conducted three times per week for 12 weeks. The participants were instructed not to change their lifestyles. The training was conducted under staff supervision. Management of the training protocol was performed by the facility staff, who created a calendar at the start date and recorded the date and time of training performed by each participant. The control group was instructed to continue normal daily activities and not to do any special exercise.

The participants were explained the purpose of the study and the methods used during the distribution of questionnaires. Participants provided individual written informed consent during the collection of the questionnaires. This study was approved by the Ethics Committee of Kansai Medical University (approval number: 1609).

### Study protocol

**Fig. 1:**
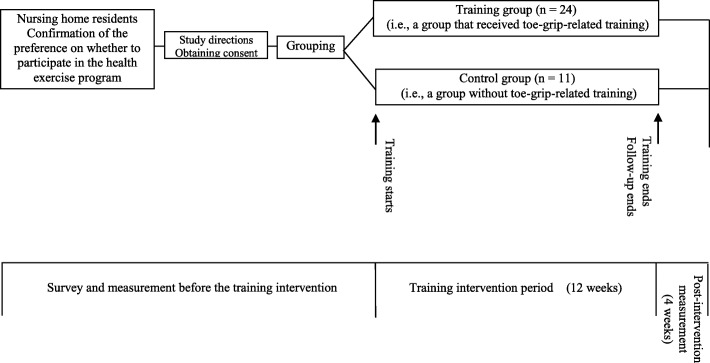
Outline of the study protocol. Study directions were orally explained to all expected participants and written informed consent was obtained. The participants were divided into two groups, according to whether they wished to participate in the toe grip-related training

**Fig. 2:**
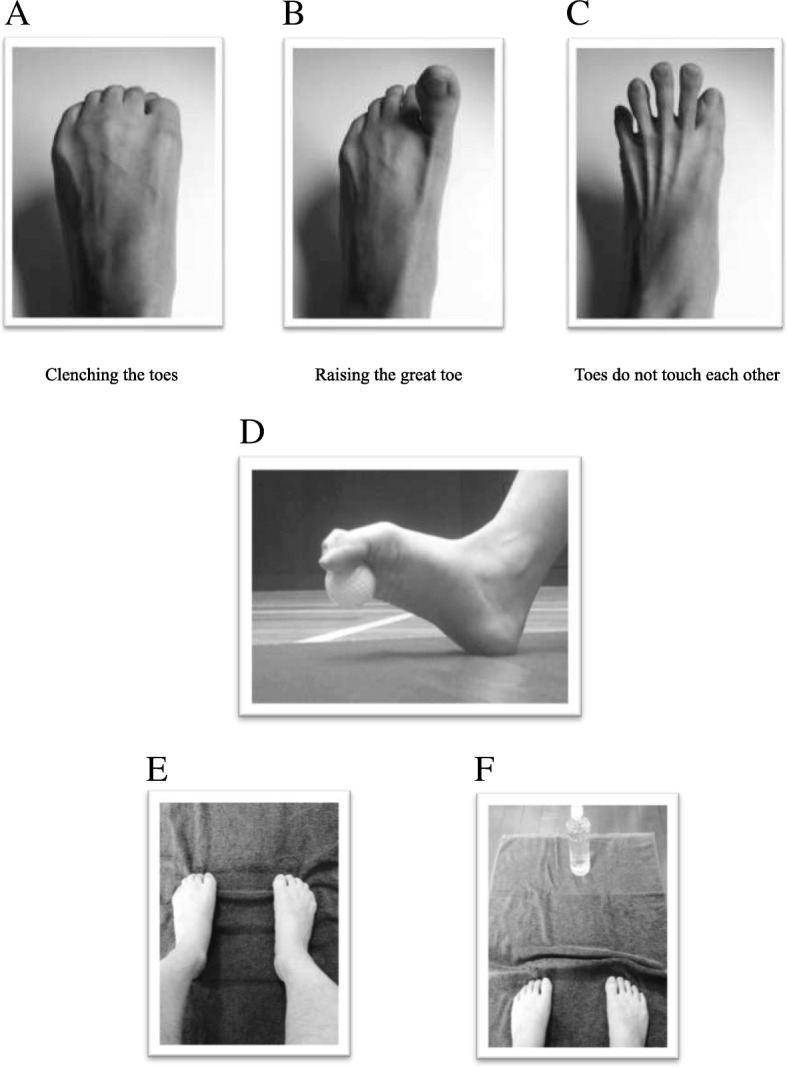
Rock-paper-scissors movements performed using the toes (**a** clenching the toes, **b** raising the big toe, **c** keeping toes apart, so that they do not touch each other). The participants practiced making rock, paper, and scissors shapes with their toes. They were instructed to perform three sets of 10 repetitions of this technique. **d** Rolling, grasping, and releasing a golf ball. The image shows a gold ball being gripped between the toes. The participants were instructed to place a golf ball on the floor beside the heel, then put their weight on it, slide their foot on it, and then to roll it with their sole to stimulate the muscles of the toes and sole. They were also instructed to grip and release the golf ball repeatedly using their toes. They performed the training for a total of 5 min. Towel-gathering exercise: the participants were instructed to gather a towel with their toes without lifting the toes from the floor. This training was performed three sets using 1-m towel in the sitting position. **e** The feet are placed on the towel. The toes are used to gather the towel towards the participant. **f** A 500-mL PET bottle filled with water was placed on the end of the towel as a load

The study protocol is shown in Fig. 1. This was a single-center non-randomized controlled study. Participants were subsequently divided into two groups: the training group (i.e., the group that received toe grip-related training) and the control group (i.e., the group that was instructed to lead a totally normal life without toe grip-related training). Baseline endpoints were evaluated, and the effects of toe grip-related training were examined following a 12-week intervention (Fig. 2). The primary outcome was changes in the toe grip strength, and the secondary outcomes were changes in other variables.

### Endpoints

#### Measurement of toe grip strength

**Fig. 3:**
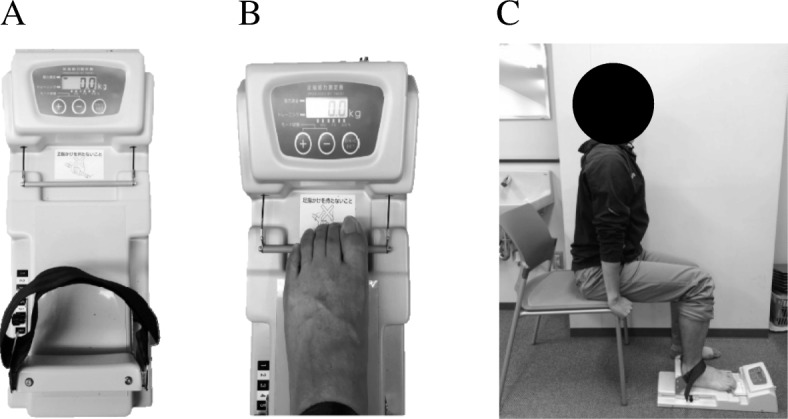
Measurement of toe grip strength: **a** A toe grip dynamometer (T.K.K. 3361; Takei Scientific Instruments, Niigata, Japan) used to measure toe grip strength. **b** The grip bar of the instrument was adjusted to the first metatarsophalangeal joint of the participant. **c** The participants sat on the edge of their seat keeping their trunk in a vertical position, with both hands holding the chair and the hip and knee joints bent to approximately 90°

Measurement of toe grip strength is shown in Fig. 3. A toe grip dynamometer (T.K.K. 3361; Takei Scientific Instruments, Niigata, Japan) was used to measure toe grip strength. The reliability of this instrument has been previously reported (Fig. 3a) [18]. The usefulness of the equipment in clinical settings and field research for people aged 20–79 years has been established [18, 19]. The substantial intra-rater and inter-rater reliabilities of the toe grip dynamometer, based on the criteria by Landis and Koch, indicate that it is suitable for clinical use [20]. The intraclass correlation coefficients (ICCs) of the toe grip dynamometer are 0.97 (95% CI 0.93–0.99) [21]. The minimal change detectable using this instrument was shown to be 0.95 kg in our previous study [5]. Before measurement, patients were asked to practice several times. Then, we performed the measurement on both sides in duplicate and calculated the mean values of the better scores.

In order to perform a measurement, the grip bar of the instrument was adjusted to the first metatarsophalangeal joint of the participant (Fig. 3b). The participant sat on the edge of their seat keeping their trunk in a vertical position, with both hands holding the chair, with hip and knee joints bent to approximately 90° (Fig. 3c). The measurement was performed twice, on both right and left sides, after a few practice measurements. The highest values obtained were used for data analysis. Assessment was performed by two health fitness programmers.

#### Mini-Mental State Examination

Cognitive function was assessed using Mini-Mental State Examination (MMSE; point). The MMSE cutoff score is 23/24 points for cognitive dysfunction and 27/28 points for mild cognitive impairment [22, 23]. The maximum MMSE score is 30 points. The lower the score is, the higher the severity of cognitive disorders. A score of 20 to 24 suggests mild dementia: in this study, the cutoff for suspected dementia was set between 20 and 21. One-to-one interviews were conducted by survey staff using the MMSE questionnaire.

#### Fall Risk Index

Fall risk was assessed using the Fall Risk Index (FRI; point). The FRI is a fall risk prediction table consisting of 21 items (8 items covering physical function; 7 items studying cognition, sensory organs, and locomotive organs; 1 item looking at medication; and 5 items examining environmental factors) for early detection of a fall risk [24]. One-to-one interviews were conducted by survey staff using the FRI.

Physical function endpoints were measurement of toe grip strength, the toe skill test (TS), standing on one leg with eyes open, the 10 times sit-to-stand test (SS-10), the timed up and go (TUG) test, and the 5-min gait speed test.

#### Toe skill test

The toe skill test is shown in Fig. 3. In the toe skill test, the participants performed rock-paper-scissors movements using their toes. Flexion of all the toes and the ability to oppose the hallux were examined using the “rock” and “scissors” movements, respectively. Toe abduction of both feet and the ability to spread the toes without them touching each other were examined using the “paper” movement. The participants were given 1 point for each successful trial, with a maximum of 3 points possible. Assessment was performed by two health fitness programmers.

The evaluation of other physical fitness is shown in Table 2.

**Table 2:** Measurement sites

Site	Measurement method
Standing on one leg with eyes open [25]	The participants put their hands on their waist and stood on one leg with eyes open. The participants were performed twice and the highest score was recorded. Maximum value was 120 s.
10 times sit-to-stand test [26]	This measurement started with standing using a 40-cm chair. The elapsed time of 10 trials of the sit-to-stand movement was recorded.
Timed up and go [27]	The distance between the edge of the participant’s chair and the distant edge of a mini-cone was 3 m. The participants were instructed to check the position of the chair before sitting down.
5-m gait speed test [28]	The maximum speed reached by participants. The time required for the foot to cross the goal line during the swinging phase was recorded.

### Statistical analysis

All data are presented as mean ± standard deviation (SD) or 95% confidence interval (CI). Changes (Δ) were calculated as differences between pre- and post-interventions. The minimal detectable change (MDC) was calculated by multiplying the standard error of measurement (SEM) by 1.96 to correspond to the 95% confidence interval and the square root of 2 to adjust for sampling from baseline score. The distribution-based minimal clinically important difference (MCID) was estimated using the Cohen effect size benchmark. An effect size of 0.5 (i.e., SD of the baseline score) indicated an important change and was used as the MCID in this study. We performed the Shapiro-Wilk test to examine normality. The Mann-Whitney *U* test and the chi-square test were performed to analyze comparisons of characteristics at baseline, change in variables between the training group and the control group. The Wilcoxon signed-rank test was used for pre and post comparisons. Spearman’s rank correlation coefficient was used to analyze the correlation between Δ toe grip strength and Δ MMSE as well as Δ physical function. A stepwise multiple regression analysis was used for multivariable analysis, in order to examine independent predictors of Δ MMSE. IBM SPSS version 21.0 (IBM Co., Armonk, NY, USA) was used for statistical analysis. A *p* value of < 0.05 (two-tailed) was considered statistically significant.

## Results

### Comparison of baseline characteristics of participants

Table 3 shows the baseline characteristics for the training group and the control group. No significant differences were observed in any factors between the two groups.

**Table 3:** A comparison of baseline values between the training group and the control group

	All	Training group	Control group	*p* value
MMSE (point)	22.7 ± 5.3	23.3 ± 5.9	21.2 ± 3.4	0.053
Fall Risk Index (point)	8.6 ± 3.1	8.1 ± 2.1	9.7 ± 4.5	0.519
Toe grip strength (kg)^a^	4.5 ± 2.2	4.6 ± 2.5	4.1 ± 1.5	0.845
TS (left foot) (point)	1.3 ± 0.8	1.3 ± 0.9	1.5 ± 0.5	0.645
TS (right foot) (point)	1.3 ± 0.7	1.2 ± 0.7	1.5 ± 0.5	0.377
One-leg standing with eyes open (s)^a^	9.0 ± 11.6	10.0 ± 11.4	6.8 ± 12.2	0.160
SS-10 (s)	17.9 ± 6.4	16.7 ± 6.5	20.5 ± 5.7	0.102
TUG (s)	10.9 ± 5.7	9.7 ± 3.9	13.6 ± 8.0	0.079
5-m gait speed (s)	5.2 ± 2.3	4.9 ± 1.8	6.0 ± 3.2	0.444

Data are presented as mean ± SD

The statistical analysis used in the Mann-Whitney *U* test

*MMSE* Mini-Mental State Examination, *TS* toe grip strength test, *SS-10* 10 times sit-to-stand test, *TUG* timed up and go

^a^The value is an average of scores from both legs

### Intervention

The training group showed significant improvements in MMSE (23.3 ± 5.9 to 24.9 ± 5.3 points, *p* = 0.024), FRI (8.1 ± 2.1 to 6.8 ± 2.3 points, *p* = 0.003), toe grip strength (4.6 ± 2.5 to 6.5 ± 2.3 kg, *p* < 0.001), toe grip strength test (TS) (left foot) (1.3 ± 0.9 points to 1.9 ± 0.7 points, *p* = 0.009), and TS (right foot) (1.2 ± 0.7 to 1.8 ± 0.8 points, *p* = 0.003). In addition, SS-10 scores decreased significantly in both the training group (16.7 ± 6.5 to 19.1 ± 8.7 s, *p* = 0.014) (Table 4) and the control group (20.5 ± 5.7 to 27.1 ± 6.4 s, *p* = 0.013). The MDC was 2.5 points in MMSE, 1.0 kg in toe grip strength, and the MCID was 2.6 points in MMSE, 1.1 kg in toe grip strength at baseline.

**Table 4:** Measurements performed on the two groups

	Training group			Control group		
	Pre-intervention	Post-intervention	*p* value	Pre-intervention	Post-intervention	*p* value
MMSE (point)	23.3 ± 5.9	24.9 ± 5.3	0.024	21.2 ± 3.4	20.2 ± 4.4	0.181
Fall Risk Index (point)	8.1 ± 2.1	6.8 ± 2.3	0.003	9.7 ± 4.5	9.5 ± 3.6	0.833
Toe grip strength (kg)^a^	4.6 ± 2.5	6.5 ± 2.3	< 0.001	4.1 ± 1.5	3.9 ± 1.6	0.878
TS (left foot) (point)	1.3 ± 0.9	1.9 ± 0.7	0.009	1.5 ± 0.5	1.5 ± 0.5	0.564
TS (right foot) (point)	1.2 ± 0.7	1.8 ± 0.8	0.003	1.5 ± 0.5	1.5 ± 0.5	1.000
Standing on one leg with eyes open (s)^a^	10.0 ± 11.4	9.9 ± 15.2	0.932	6.8 ± 12.2	4.6 ± 5.0	1.000
SS-10 (s)	16.7 ± 6.5	19.1 ± 8.7	0.014	20.5 ± 5.7	27.1 ± 6.4	0.013
TUG (s)	9.7 ± 3.9	10.2 ± 5.9	0.615	13.6 ± 8.0	13.5 ± 5.6	0.790
5-m gait speed (s)	4.9 ± 1.8	4.6 ± 1.6	0.415	6.0 ± 3.2	6.5 ± 3.9	0.657

Data are presented as mean ± SD

The statistical analysis used in the Wilcoxon signed-rank test and the Mann-Whitney *U* test

*MMSE* Mini-Mental State Examination, *TS* toe grip strength test, *SS-10* 10 times sit-to-stand test, *TUG* timed up and go

^a^The value is an average of scores from both legs

Post-intervention in both groups showed significant differences in MMSE (24.9 ± 5.3 to 20.2 ± 4.4 points, *p* = 0.005), FRI (6.8 ± 2.3 to 9.5 ± 3.6 points, *p* = 0.035), toe grip strength (6.5 ± 2.3 to 3.9 ± 1.6 kg, *p* = 0.002), SS-10 (19.1 ± 8.7 to 27.1 ± 6.4 s, *p* = 0.006), and TUG (10.2 ± 5.9 to 13.5 ± 5.6 s, *p* = 0.047).

Comparisons of changes in scores collected at baseline and following intervention between the two groups are shown in Table 5. The training group showed significant increases in Δ MMSE [1.6 (0.2–3.0) to − 1.0 (2.6–0.6) points, *p* = 0.020], Δ toe grip strength [1.9 (1.2–2.6) to − 0.2 (− 1.4–1.0) kg, *p* = 0.005], and Δ TS (right foot) [0.6 (0.2–1.0) to 0.0 (− 0.3–0.3) points, *p* = 0.017] than the control group.

**Table 5:** Comparison of changes between the training group and the control group

	Δ training group	Δ control group	*p* value
MMSE (point)	1.6 (0.2–3.0)	− 1.0 (2.6–0.6)	0.020
Fall Risk Index (point)	− 1.4 (−2.2 to − 0.6)	− 0.2 (− 2.6–2.3)	0.075
Toe grip strength	1.9 (1.2–2.6)	− 0.2 (− 1.4–1.0)	0.005
TS (left foot) (point)	0.6 (0.1–1.0)	0.1 (− 0.3–0.5)	0.104
TS (right foot) (point)	0.6 (0.2–1.0)	0.0 (− 0.3–0.3)	0.017
Standing on one leg with eyes open (s)^a^	− 0.2 (− 5.7–5.3)	− 2.2 (− 11.0–6.5)	0.749
SS-10 (s)	3.2 (1.0–5.3)	6.5 (2.2–10.9)	0.102
TUG (s)	0.8 (− 1.6–3.1)	− 0.1 (− 3.4–3.3)	0.972
5-m gait speed (s)	− 0.2 (− 0.6–0.2)	0.4 (− 0.9–1.8)	0.557

Data are presented as mean (95% CI)

The statistical analysis used in the Mann-Whitney *U* test

*MMSE* Mini-Mental State Examination, *TS* toe grip strength test, *SS-10* 10 times sit-to-stand test, *TUG* timed up and go

^a^The value is an average of scores from both legs

### Correlations and multivariate analyses of Δ toe grip strength and Δ physical function

The correlations between Δ toe grip strength and Δ physical function are shown in Table 6. Δ toe grip strength showed a significant positive correlation with Δ MMSE (*r* = 0.415, *p* = 0.013). In contrast, there was no significant correlation between Δ toe grip strength and other items.

**Table 6:** Correlations between Δ toe grip strength and Δ cognitive and physical function

	Δ toe grip strength	
	Correlation coefficient	*p* value (two-tailed)
Δ MMSE	0.415	0.013
Δ Fall Risk Index	− 0.272	0.114
Δ TS (left foot)	0.102	0.562
Δ TS (right foot)	0.120	0.491
Δ one-leg standing with eyes open	− 0.226	0.193
Δ SS-10	− 0.300	0.079
Δ TUG	− 0.013	0.940
Δ 5-m gait speed	0.177	0.309

The single correlation analysis used in Spearman’s rank correlation coefficient

*MMSE* Mini-Mental State Examination, *TS* toe grip strength test, *SS-10* 10 times sit-to-stand test, *TUG* timed up and go

Thus, a stepwise multiple regression analysis was used to identify the factors for Δ cognitive function. The multiple regression analysis was performed using Δ MMSE as a dependent variable, and Δ toe grip strength, Δ SS-10, and adjustment factors (including age, gender, body mass index, the rate of certification of long-term care insurance, and cerebrovascular disease) as independent variables. The results identified toe grip strength as the only independent risk factor (unstandardized coefficients 0.639, standardized coefficients 0.395, 95%CI 0.113–1.166, *r*^2^ = 0.131, *p* < 0.05) (Table 7).

**Table 7:** Stepwise multiple regression analysis of the changes in cognitive function

	Standardized coefficients	*p* value	Tolerance	VIF
	β			
Age	− 0.120	0.461	1.000	1.000
Body mass index	− 0.001	0.996	0.946	1.057
Gender	0.260	0.117	0.935	1.070
The rate of certification of long-term care insurance	− 0.108	0.510	0.982	1.019
Cerebrovascular disease	− 0.011	0.946	0.993	1.007
Δ toe grip strength	0.395	0.019	1.000	1.000
Δ SS-10	0.111	0.513	0.922	1.084

Adjustment factors were age, body mass index, sex, the rate of certification of long-term care insurance, cerebrovascular disease, and Δ SS-10

The multivariable analysis used in a stepwise multiple regression analysis

*SS-10* 10 times sit-to-stand test

## Discussion

The results of this study have established our hypothesis. The 12-week toe grip-related training program, which was performed three times per week, significantly improved MMSE score, FRI, toe grip strength, and TS. The control group was instructed to lead a totally normal life without performing any special exercise. Although there were significant differences in both Δ MMSE and Δ toe grip strength between the two groups, the value of SS-10 significantly decreased in both groups. To our knowledge, this is the first study to demonstrate that toe grip training improves cognitive function and toe grip strength in nursing home residents. There were no occurrences of trauma or injury during the study period.

In addition, there was a significant correlation between Δ toe grip strength and Δ MMSE. The results of the multivariable analysis, following adjustment for age, body mass index, gender, the rate of certification of long-term care insurance, and cerebrovascular disease, still identified Δ toe grip strength as the only independent factor of Δ MMSE. It is believed that cognitive function can be improved by the effects of conscious toe grip training on factors including cerebral metabolism and cranial nerve activity, when the toes are not frequently exercised [15]. Thus, we found a whole-body coordination in these populations. Voluntary exercises are required because the toes are not exercised on a daily basis. In addition, it has been reported that performing toe exercises increases peripheral somatosensory input and cerebral blood flow in the primary somatosensory area [29, 30]. Training intervention for the toes, which are not routinely used, may improve cognitive function via an increase in the rate of cerebral blood flow. Based on the above, it is thought that toe grip training may contribute to an improvement in cognitive function. In the single variable correlation analysis of Δ toe grip strength, only Δ MMSE was significantly correlated. However, we considered that because this analysis took into account only two variables, it needs to be adjusted for age and gender, and thus, we used the stepwise multiple regression. In addition, Δ toe grip strength was shown to be an independent predictor of Δ MMSE in the present model, but only 13% of the variance was explained and the presence of other influencing factors is expected.

The MDC and MCID of toe grip strength were 1.0 and 1.1 kg at baseline in this study, respectively. In this study, the change in toe grip strength was identified to be meaningful at 1.9 kg. Conversely, because the MDC and MCID of MMSE were 2.5 and 2.6 points, respectively, at baseline in this study, the possibility of measurement error remains, and the clinical significance of improvements of 1.6 points was unclear. It was however an interesting finding that warrants further investigation. The change in MMSE between the two groups showed a significant difference, and the MMSE in the non-intervention groups did not show a significant change (range − 0.1 to 0.7 points) in the 12-week intervention study for the elderly [31, 32]. Thus, we believe that a change of 1.6 points is induced by toe grip training and has important physiological implications. At least, toe grip training had an effect of toe grip strength and toe skill, and toe grip strength was an independent factor of change in MMSE (Table 6).

On the other hand, toe grip training did not improve predictors of physical performance other than toe grip strength, and SS-10 scores decreased significantly in both groups. The toe grip training performed in this study seems to have improved the ability to press the toes downward and grip with the toes by directly affecting them. The activity of the flexor digitorum longus promotes the simultaneous contraction of the leg muscle and the muscles surrounding the ankle joint and is involved in multi-joint functions [1–5]. The reason that static or dynamic balance and gait function did not improve in this study may be that the contraction of the flexor digitorum longus causes the functional chain movement of the lower extremities through indirect effects. In other words, toe grip training alone is not sufficient to improve locomotive performance in nursing home residents [16, 17]. Toe grip training needs to be combined with a conventional aerobic or resistance training program [33]. Toe grip training is a safe and convenient training method that can be performed at any time (for example, while watching TV) and anywhere (for example, in the bed or bath).

This study is unique in that it examined the effects of toe grip training in nursing home residents. The participants in this study were elderly individuals, with high ADL, who lived in a home for the elderly. One of the problems with exercise intervention in the elderly is the low rate of continuation of exercise programs. In this study, with the support of the facility staff, the exercise continuation rate was high (85.7%). The reason for the high exercise continuation rate and exercise effects may be that the facility staff that was familiar with participants provided the instructions for the toe grip training. Toe grip training is an easy and safe method that may help prevent falls and dementia. This technique is a useful new method in the field of long-term preventative care.

In this study, the subjects divided themselves into the two groups. As members of the TG group had decided for themselves to participate in this training, it is possible that they were more highly motivated than members of the CG group. In real life, in most care homes, participation in activities is voluntary. It is thus likely that the intervention in this study reflects the way that people live and act in actual institutions. The fact that participation was voluntary also generated some variation in sample size, but there were no significant differences in age, sex, or MMSE at baseline. Nevertheless, the fact that members of the TG group were younger and included more men than the CG group may have contributed to differences in baseline values. For example, the mean BMI of the CG group was significantly lower, suggesting that it may have been affected by age and sex ratio. There was also a two-point difference in baseline MMSE and differences in cognitive ability which may contribute to the aging process [34]. Over the short term, however, activities of daily living and regular exercise interventions have a greater effect on the body composition and cognitive abilities of elderly people than age or sex [35]. The possibility that allowing the subjects to allocate themselves to each group may have resulted in more active subjects congregating in the intervention group cannot be excluded. However, this is a reflection of real life. The changes between the two groups thus convey an important message which is a significant physiological anthropological result.

This study has a number of limitations that should be discussed. First, the participants were divided into two groups according to their personal preference. Although most participants in the training group were expected to be motivated and active in a daily life and in the nursing home, this study did not assess physical activity in their daily lives. In previous studies reporting significant associations between physical activity and cognitive function in the elderly [36], both groups were instructed to lead a normal life without performing any special activities during the intervention period. Thus, cognitive function and physical performance may not have been affected by physical activity in these people. There was no significant difference in the participants’ characteristics or physical function at the baseline values. Since all participants lived in a single facility, physical/human environments and meals were controlled. In addition, confounding factors are considered to have been adjusted by using multivariate analysis. Second, the physical activity in elderly nursing home residents varies widely depending on the facility. Thus, to exclude the difference, we performed this study in residents of a single facility. Finally, the MMSE scores in this study were somewhat low. The validity of the questionnaire survey in this study is guaranteed because the survey interviews were conducted on a one-on-one basis. Lastly, this study showed that toe grip strength and MMSE were significantly improved by training and the independent factor of ΔMMSE was Δ toe grip strength.

## Conclusions

Toe grip training increased the toe grip strength, toe skill, and cognitive function. Furthermore, change in cognitive function was associated with change in toe grip strength.

## Data Availability

The datasets used and analyzed during the current study are available from the corresponding author on reasonable request.

## Abbreviations

FRI: Fall Risk Index
MMSE: Mini-Mental State Examination
SS-10: 10 times sit-to-stand test
TS: Toe grip strength test
TUG: Timed up and go
